# Being Irrationally Funny as a Cognitive Psychologist: Interview With Dan Ariely

**DOI:** 10.5964/ejop.v11i4.1083

**Published:** 2015-11-27

**Authors:** Dan Ariely, Beatrice Popescu

**Affiliations:** a Duke University, Social Science Research Institute, Durham, NC, USA; b Faculty of Philosophy, University of Bucharest, Bucharest, Romania

**Keywords:** cognitive psychology, irrational behavior, behavioral economics, trauma, resiliency

## Abstract

The idea of interviewing Dan Ariely was somehow latent on my mind since I started being interested in cognitive psychology and cognitive behavior psychotherapy, but actually got more ardent ever since irrationality became a research topic for his team at Duke University. I picked him as an interviewee thinking not only at his exceptional skills as a researcher and as Kahnemann ‘disciple’, but mainly for his fantastic wit, true modesty and utmost interest in making people’s lives easier and more comfortable, by creating awareness on a lot of topics otherwise neglected. Dan Ariely’s very agreeable personality and humor would not let you think of him as a burnt casualty who, in his youth struggled to survive a personal drama, so well-documented in his paper “Painful lessons” posted on the MIT website (http://web.mit.edu/ariely/www/MIT/Papers/mypain.pdf). I think reading his paper and also this transcribed interview with him would be also comforting for people who found out about Bucharest fire incident that rocked our society and also for people who are personally related to this tragedy.

**Beatrice Popescu:** First of all, dear Professor Ariely, we would like to thank you for squeezing this interview among the huge amount of activities you are currently involved in. You have started your BA studies with hard sciences, mathematics and physics at Tel Aviv University and you continued them with philosophy and psychology. How did this switch happen and when did you decide to embrace cognitive psychology as a research adventure?

**Dan Ariely:** Hello, Bucharest, sorry for the delayed response, hopefully this will be useful. How did the switch happen? Actually the switch happened not because I was thinking about it, but just because I was kind of forced to switch. So basically what happened was that I was badly injured many years ago when I started studying at the university, when I was still suffering from a lot of pain and I had a lot of challenges and among my many injuries, one is that my hands don’t function very well and it’s hard for me to move them. One of the things that happened was that I had to write to the university. I started studying physics, then I thought I could do math and physics without writing, which turned out to be wrong, but, because I couldn’t write, I had to pick something else and I picked philosophy and psychology, which allowed me to actually not use my hands as much and then, even though it started by their requirement, very quickly I fell in love with psychology, particularly with experimental science. It can happen largely by accident, because one of my first professors, Hannan Frenk, he was injured himself, he lost both of his legs in an explosion and he kind of turned his personal interest in pain into his professional interest. Through him I kind of learned a lot about experimental psychology but I also learned about how you can take your on interest in life and turn it into your professional interest.

**Beatrice Popescu**: Your mentor is Daniel Kahneman, the famous cognitive psychologist who also recommended you to enter the field of behavioral economics and urged you to also obtain a doctorate in business administration. Can you describe in a few phrases in which way your collaboration with Prof. Kahneman changed your thinking?

**Dan Ariely:** Daniel Kahneman is an incredibly thoughtful, interesting, creative guy and he has influenced me in many ways. He’s influenced me in the way that I thing about collaborations, in the way that I think about students, in the way that I think about the people I work with, in terms of the topics I work on. In the last years Danny has moved to try and influence policy in a bigger way and that has influenced me as well and I think actually the whole field is now much more interested in contributing to the questions about how do we design life in a better way from a policy perspective.

**Beatrice Popescu**: I must confess, your paper “Painful lessons” posted on the MIT website moved me a lot, I consider it one of the few well researched ‘pain and suffering’ documented descriptions and also an important lesson of stoicism in a hedonistic world. As a research psychologist, what advice do you have for people hit by drama in their lives?

**Dan Ariely:** Thanks for the question. This question is about my essay on pain. What advice do I have for people who are hit by trauma? I actually don't know. As you can imagine, lots of people with injuries write to me and they ask me a lot of interesting questions, tell me about their lives, they ask questions about resiliency, about what gets people to be more resilient, less resilient? Resiliency is something that I am deeply interested in and we actually have some research projects on this right now, but where really resiliency comes from, we don’t know exactly: there are some personality traits of course that are related, there is the question about feeling of hope and not feeling helplessness, there is the question of having a supportive family, having an optimistic view of the future, all of those are true but to deeply understand resilience, we're not there yet, but this is something that I'm certainly interested in and trying to explore more.

**Beatrice Popescu:** Your book “Predictably Irrational” presents experimental evidence that proves yet again that human behavior is irrational and exposes some of the irrational behaviors we engage in. Is behavioral economics able to explain all of our behaviors and also, does it have an answer for all our worries?

**Dan Ariely:** No, absolutely not, we can explain some of them and they have an answer for those and again, of course not. Two things are important to consider here: the first one is that, of course, science is just kind of work in progress, but the other thing to realize is that the world is also changing. Therefore, if you think about the world of physics, the world of physics is kind of stable, but if you think about the world of social science, the world of social science keeps on changing: ten years ago we didn't have cell phones, we didn't have facebook and life was different, the patients were different, the mistakes that people were making were different. I think that social science would never get all the records of all the irrational behaviors, because technology and society would keep on moving forward and people would discover new ways to get things wrong or they will discover some ways that were dormantly wrong to be now more actively wrong.

[Contrib contnr1]**Beatrice Popescu:** Seeing the amount of free downloads on the web despite all copyright laws, from movies to books and research work, how do you think we can change this situation? Can we possibly cultivate moral behavior in people by teaching ethics or ethical behavior starting from gymnasium? Do you think offering products at a discounted price would curb the free download phenomenon?

**Dan Ariely:** There's lots of free downloads on the way people download a lot of stuff illegally. Can we do it by getting people to be more moral and can we discount prices sufficiently to curb the frees? I don't think we could, I don't think either of them. I think that what we find about this, honestly, is that people find lots of ways to rationalize their behaviors and I think that when it comes to digital goods people have lots of good ways, lots of effective ways to rationalize their behavior. They can say things like: “nobody's going to really be hurt” and “nobody's really suffering” and “I wouldn't do it anyway the other way around” and “the people are making too much money”, so people can say all kinds of stories to themselves and I don't think it's about price. Even if it was very cheap, they would acknowledge it was very cheap, but they would say yes, it could be a little bit less, but I don't think it could stop it.

**Beatrice Popescu:** Together with a team of researchers in 2008 you won the Ig Nobel prize for your research paper on the behavioral impact of a higher price. One of the study conclusions is that even though in general we look for free or discounted products, when it comes to health, we look for higher prices in medicines (and brand names) thinking that there is a positive correlation between price, quality and efficiency . Do you think this could also apply to psychotherapy fees? Could a person consider for example that if offered pro bono, the chances are that a therapy does not work and if highly priced, it works better?

**Dan Ariely:** There are some cases in which price is high, people have high expectations and therefore they have a higher placebo effect and when the price is low, they have low expectations and therefore a low placebo effect and even a nocebo effect in that case. Do I think this could apply to other things as well? Of course, I think that the price certainly grabs our attention, we think about it and because of that, it has an ability to influence the expectations of quality. Now, here's the thing: if you take something that people know it’s quality, for example I take a very expensive computer or a very expensive phone and I tell people “I'm giving you this for free” and they know it's of the same type and quality, they are not going to say: “Oh, this must be terrible!”, but if I don't tell them about it and they don't know the quality in advance, it can backfire. And the reality is that we have lots of things in those cases when people don't know the quality.

[Contrib contnr2]**Beatrice Popescu**: In rational emotive behavior therapy (Albert Ellis) we often try to help the client to replace the irrational thinking with rational cognitions, hoping that negative emotions elicited by automatic thoughts will turn into positive or neutral ones that follow rational thoughts. In the paradigm you propose, if I understand well, a bit of irrationality is not always to be rejected. When do you think it’s reasonable to be irrational?

**Dan Ariely:** Trying to help people replace irrational thinking with rational cognitions? Yes, it's a question of what do we think of this ‘irrational’, there are lots of definitions of it. One definition is that whatever is stupid is irrational, and then of course, by definition you don’t want stupid things. Another thing is to say this is about the economic definition, when people are not selfish for example it’s irrational and that of course we don’t want to say that’s the case and we want to eliminate that, to tell people should stop helping other people or not consult their emotions and so on. I don't think it's always the case, but it’s really a question of what do we consider as irrational. My main concern with irrationality is not the economic definition. I am interested in the economic definition because if we can shape the economic definition people might not adopt economics as a tool for policy, but the part that interests me more is the part where people get things wrong and because they don’t understand what’s controlling their behavior and from that perspective I think psychologists have a lot to do in terms of educating people to better understand the real reasons for their behavior.

**Beatrice Popescu:** The (Dis)honesty Project is your latest research assignment that turned into a documentary film, presented at Montclair Film Festival. Like cruelty, dishonesty turns out to be a remarkably prevalent phenomenon apparently better explained nowadays by circumstances and cognitive processes than by concepts like character and moral development. However, since in one of your experiments in which you asked the subjects to sign an MIT ‘honor code’ the cheating did not occur at all, do you think the idea of signing and complying to Ethical Codes could possibly increase ethical behavior in humans? Do you think ethics and behavioral science could explore issues in an interdisciplinary conceptual framework in order to increase people’s awareness towards moral behavior?

**Dan Ariely:** The “Dishonesty Project” is a movie and then some things around it about how we understand dishonesty. Here is some research on dishonesty, some stories about people who were dishonest and how they deteriorated over time and what happened to them and we tried to go back and forth between those stories. And the research to try and understand how research fits with people's real lives and what are the implications of fathom. I actually think the producer Yael Melamede and the rest of the team did an amazing job in this movie and I think we need to do multiple things: we need to increase awareness and then we need to create different systems because you see, if people just become more aware, awareness will go away, at some point it will be other things that will be important so it’s not just awareness that we can rely on. But we need to combine awareness with interest in actually doing something else and then doing something else.

**Beatrice Popescu:** Procrastination is another hot topic among your research interests. The conclusions of one of your papers are that people are good at self-imposing meaningful deadline to overcome procrastination, the deadlines are effective in improving task performances, but unfortunately people cannot manage to set those deadlines optimally. Do you have a suggestion on the optimal setting of deadlines, especially for graduate students striving to finish writing their PhD thesis?

**Dan Ariely:** Procrastination is a very tough thing. Over the last few years, we actually tried to create an app that would help people manage their time and we recently sold the little company to a big company, to Google, and I am hoping that they will use it. But if you think about it, I think that the gateway for productivity is *the calendar*: you look at the calendar, you see what you have to do and then you do what the calendar tells you. If you want to write your PhD thesis, then that task needs to be on your calendar. So what you need to do is to take maybe two productive hours you have every day, maybe between 9 and 11 a.m. or maybe in the evening or maybe one on one however it is. And you should write down: this is when I'm working on my thesis and when you don't work on it, you should admit that you are not working on it. But if you basically say let me just let other things take hold of my calendar and I work on my PhD when the time comes, the time will not come. So I think that the calendar is an important gateway for good behavior.

**Beatrice Popescu:** In your TED presentations you always insert anecdotes and humorous bits that your audience simply love. Charlie Chaplin once said: “Through humor, we see in what seems rational, the irrational; in what seems important, the unimportant”. Do you agree with him?

**Dan Ariely:** Is humor important to see rational and irrational? I am not sure about ‘rational’ versus ‘irrational’, but I think humor is a way to view life. And I think through humor and jokes and fable, we can actually have a good view of human nature. And I think it's a really wonderful start to view important things. I think it also gives people a break, it gets people to be less serious about themselves, it opens people's mind, it relaxes them, it makes them a bit more creative, so all good things.

**Beatrice Popescu:** You are not only an esteemed university professor teaching in the marketing department at Duke University, but also a cognitive psychologist and behavioral economics researcher, a social media influencer, an analyst, an acclaimed speaker travelling all over the world, a fervent blogger, but also a father and a husband. When do you find time for everything? Is prioritization a difficult task for you?

**Dan Ariely:** I am not actually teaching in the marketing department anymore, I'm teaching at Duke but not in any particular department. How do I have time for everything? I don't actually, as you can tell from this interview that I'm late on it. I don't have time for everything and I actually don't sleep that much and I am very stressed. So I'm not one of those people who is managing everything, I’m one of those people who tries to, has good intentions but I fail, I fail often and time management is one of those things that is very very hard for me and to figure out priorities is very complex. I often just work as hard as I can for as long as I can, but I don't have a secret on work-life balance, it’s certainly is not one of my ‘forte’.

**Beatrice Popescu:** What is your relationship with your students at Duke University? Do they see you as a mentor or more as an erudite friend? When describing them the market realities before landing a job in the real world, do they seem challenged or discouraged?

**Dan Ariely:** What is my relationship with my students? I think that it's a collaborative relationship, I think of it more like a father when kids start or a tutor when students start. My role is to provide them with all the help that I can, to let them flourish and then as they become adults, we become more of friends. I'm still in touch with all of my past students, I think of ourselves as an extended family, I even like the students of my previous students, I feel like any of my grandchildren, so that's very much a family feeling to the experience. They know about my life, I know about their lives, once a year I try to take all my students and my family for a vacation together so everybody gets to hang out and meet each other and spend some time together, these are two sides of my life. They meet from time to time in other cases as well, but we try to also make sure we meet in those as well.

**Beatrice Popescu:** In the end of our talk, dear Professor, I would like to ask you if you have other projects in mind for the next period and also to wish you a happy well-deserved summer holiday.

**Dan Ariely:** First of all, thanks for the nice wishes for the summer holiday. In terms of other projects, my goal for the near future is to create a center that will focus on helping people make better financial decisions. I'm still not exactly sure how we'll do it, but I think that money is one of those things that technology can either help us spend more and think less, or get us to stop, reflect and behave better. So I'm hoping to be able to contribute to the behaving better. In any case, I hope this is helpful and if you have more questions, I am on my way to Brazil but I will try to answer by email. Bye.

